# The Novel Ionic Liquid and Its Related Self‐Assembly in the Areas of Energy Storage and Conversion

**DOI:** 10.1002/smsc.202200048

**Published:** 2022-08-21

**Authors:** Runtong Wang, Chengdong Fang, Le Yang, Ke Li, Kailing Zhu, Guofeng Liu, Jiajia Chen

**Affiliations:** ^1^ State Key Laboratory for Physical Chemistry of Solid Surfaces Innovation Laboratory for Sciences and Technologies of Energy Materials of Fujian Province (IKKEM) Collaborative Innovation Center of Chemistry for Energy Materials (iChem) Department of Chemistry College of Chemistry and Chemical Engineering Xiamen University Xiamen Fujian 361005 China

**Keywords:** energy storage and conversion, novel ionic liquids, self-assembly

## Abstract

Ionic liquids (ILs) are one kind of molten salts, which have been widely used across multiple disciplines in science and engineering. The recent development makes ILs no longer just as the solvent, and more attention has been put to applications of their unique structure and functionalities. This raises the importance to explore advanced IL‐derived materials, which retain most of the characteristics of ILs and are endowed with new features, namely novel ILs. The novel ILs, including deep eutectic solvents (DESs), poly(ionic liquid)s (PILs), ionic liquid crystals (ILCs), and redox‐active ionic liquids (RAILs), have distinct advantages over traditional ILs, such as enhancing the reaction rate, selectivity, and productivity in various chemical reactions. Given the unique physical and chemical properties, electrochemical behavior, and self‐assembled structures, novel ILs have emerged as promising materials in various applications, specifically in energy storage and conversion. Herein, the intrinsic properties of novel ILs and their related self‐assembly behavior in advanced energy storage technology are focused. And the perspectives and challenges of novel ILs in the fields of energy storage and conversion are also proposed.

## Graphical abstract

Novel ionic liquids with specific features, such as deep eutectic solvents, poly(ionic liquid)s, ionic liquid crystals and redox‐active ionic liquids, appear to meet the rigorous demands in different applications. This review discusses the properties and self‐assembly behavior of those novel ionic liquids, and provides comprehensive insights in the emerging uses of these characteristics in various electrochemical energy storage and conversion.

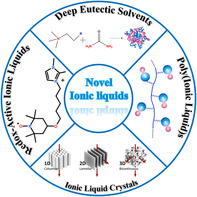

## 1 Introduction

Ionic liquids (ILs) are defined as liquids composed absolutely or almost entirely of anions and cations with melting points lower than 100 °C, which can be regarded as a special circumstance of molten salts.^[^ ^1^ ^]^ Since the first room‐temperature ionic liquid (RTIL) was found by Walden in 1914,^[^ ^2^ ^]^ ILs have been studied for more than 100 years. Compared to other liquid materials, the strong Coulombian force between anions and cations offers ILs unique properties such as ionic conductivity, low volatility, noninflammability, high chemical and electrochemical stability, and so on.^[^ ^3^ ^]^ These properties coupled with adjustable structure and functionality allow ILs to evolve from unique liquid mediums to the solvents/electrolytes widely used across multiple disciplines in science and engineering.^[^ ^4^, ^5^, ^6^, ^7^, ^8^, ^9^ ^]^

The incorporation of ILs and advanced energy storage and conversion technology seems to be a nice strategy to meet the increasing demand for clean and sustainable energy.^[^ ^10^ ^]^ However, traditional ILs based on ammonium, alkylpyridinium, dialkylimidazolium, and phosphonium cations, are generally expensive, nonbiodegradable, and toxicities, which are unfavorable to achieving the aforementioned goals.^[^ ^11^ ^]^ Thus, numerous efforts have been devoted to exploring the advanced ILs‐derived materials, which retain most of the characteristics of ILs and are endowed with new features. Novel ILs, including deep eutectic solvents (DESs),^[^ ^12^, ^13^ ^]^ poly(ionic liquid)s (PILs),^[^ ^14^, ^15^, ^16^ ^]^ ionic liquid crystals (ILCs),^[^ ^17^, ^18^ ^]^ and redox‐active ionic liquids (RAILs),^[^ ^19^, ^20^ ^]^ have been synthesized and have been proved to be promising materials to compensate for the inadequacies of the traditional ILs.

In general, those four types of novel ILs exhibit unique features and advantages in the fields of energy storage and conversion, respectively. For example, DESs are inexpensive, biodegradable, and nontoxic, they have great advantages in building environmental‐friendliness energy storage devices.^[^ ^21^, ^22^ ^]^ The PILs have a polymeric structure while maintaining ionic conductivity. This means they are excellent potential materials for preparing binder, membranes, and solid‐state electrolyte.^[^ ^23^ ^]^ They are also a kind of building blocks for self‐assembly. ILCs with enhanced ordered structure and unique phase behavior are able to achieve the efficient and directional conduction of species, which are excellent properties to construct high‐performance energy storage devices.^[^ ^24^ ^]^ Besides, RAILs are a liquid material with ionic conductivity and redox centers and have exhibited promising potentiality for use as redox additives and electroactive electrolytes.^[^ ^25^ ^]^

In this review, we focus on the intrinsic properties of novel ILs and their related self‐assembly behavior for constructing high‐performance energy storage and conversion devices. In the following four chapters, we discuss the unique properties, structure, and self‐assembly behavior of DESs, PILs, ILCs, and RAILs, and further discuss how the special features can be incorporated into advanced energy storage and conversion technology. Meanwhile, outlook and perspectives are proposed for future strategies to develop advanced novel ILs and promote the incorporation of advanced energy storage technology.

## 2 DESs

**Figure 1:**
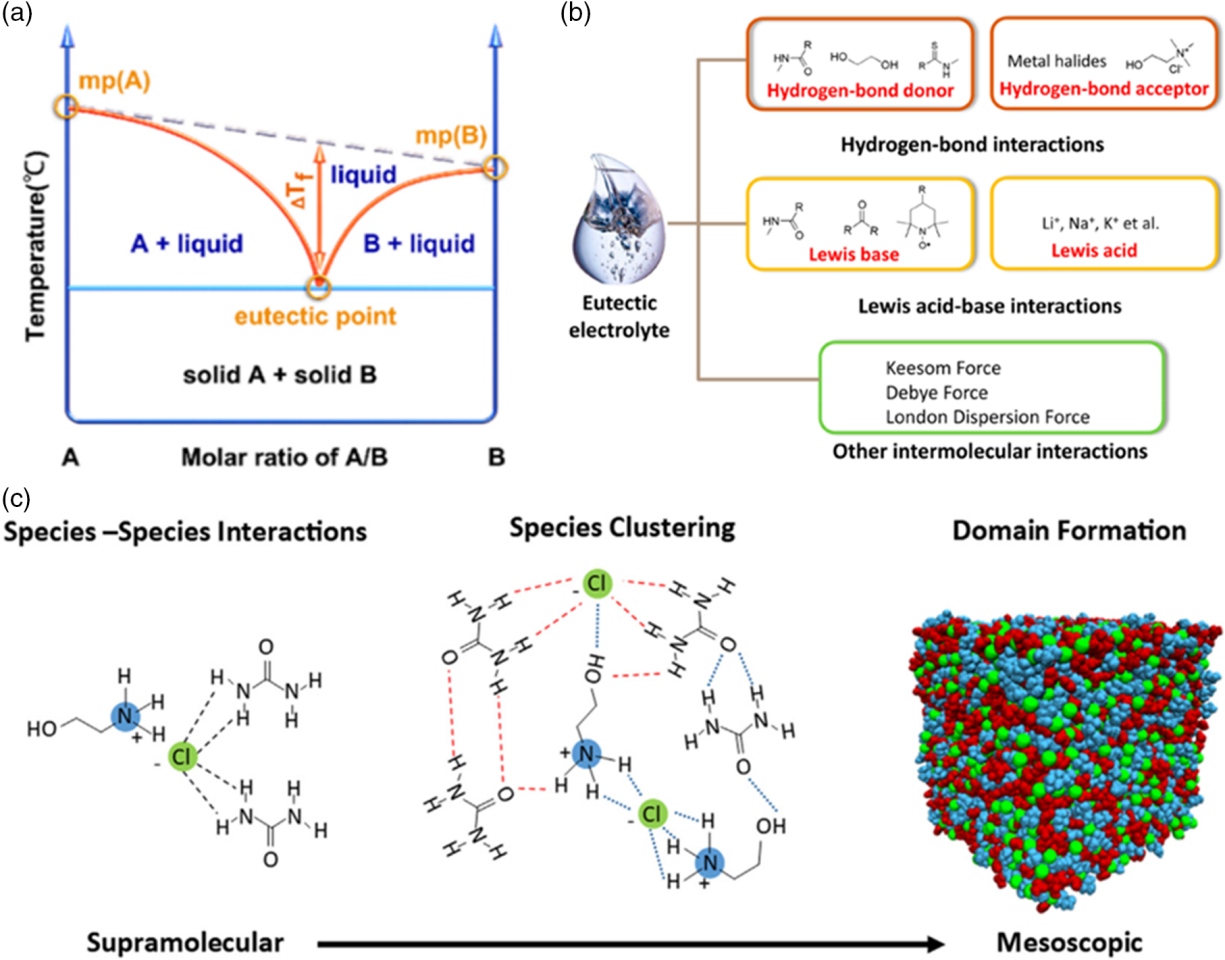
The formation mechanisms and interactions. a) The general phase diagram of deep eutectic solvent (DES) with two components. Reproduced with permission.^[^ ^12^ ^]^ Copyright 2022, American Chemical Society. b) Various intermolecular interactions for influencing the information and properties of DESs. Reproduced with permission.^[^ ^28^ ^]^ Copyright 2020, American Chemical Society. c) The structure of DESs from supramolecular to mesoscopic. Reproduced with permission.^[^ ^29^ ^]^ Copyright 2021, Elsevier B.V.

The natural crystallization tendencies of the component would be obstructed by the mixing of other components, which results in a dramatic decrease in the phase diagram (**Figure** **1**a). The liquids with this property have been called “DES,” which was first reported by Abbott et al. in 2003.^[^ ^26^ ^]^ Compared to traditional ILs with a complex preparation process, DESs are prepared by simply heating and stirring the constituents of the DES without additional solvent, other chemical reactions in the traditional sense, or even purification steps.^[^ ^22^, ^27^ ^]^ The biodegradable and nontoxic constituents and more economical preparation method allow DESs to be promising alternatives to traditional ILs. In addition, DESs can exhibit many different properties and thermodynamic behavior. For instance, many DESs tend to supercool in the conventional cooling process, and even undergo a glass transition instead of phase change at the extreme cooling process, which are greatly different from common solvents/electrolytes.^[^ ^13^ ^]^ The prevailing opinions hold that the properties and nanostructure of DESs are dominated by three types of interactions, including hydrogen‐bond interactions, Lewis acid–base interactions, and van der Waals interactions (Figure 1b).^[^ ^28^ ^]^ Hydrogen‐bond interactions are suspected to be the key driver of the formation of DESs and the dramatic decrease of melting point. The extensive hydrogen bonds lead to the presence of a hydrogen bond network and cause the charge delocalization, which has great influences on the features. In addition, Lewis acid–base interactions and van der Waals interactions are also believed to play an efficient role in the formation of DESs and the adjustment of the coordination state.

The complex molecular and ionic interactions will also induce the unique nanostructure. To date, numerous attempts have been carried out to reveal the nanostructure of DESs by using neutron diffraction,^[^ ^30^ ^]^ empirical potential structure refinement (EPSR), and theoretical calculation.^[^ ^31^ ^]^ The resultant data has provided some insights that the DESs appear as self‐assembled materials with distinct, well‐defined order and heterogeneous nanostructure.^[^ ^29^ ^]^ The clustering of species could be observed at nanoscales, and the propagation of clustering leads to the domain formation at larger length scales (Figure 1c). It is obvious that the strength of the intermolecular interactions plays an important role in influencing the nanostructure, and the physical and chemical properties. For example, Migliorati et al. used molecular dynamics simulations to analyze and compare the structural differences between a 1:2 mixture of choline chloride and urea and a 1:2 mixture of butyltrimethylammonium chloride and urea. The presence of a hydroxyl group on choline chloride led to a more complex hydrogen bond network than butyltrimethylammonium, the interaction between chloride and cation had been weakened. These phenomena caused a different motif of cage‐like structures, which could be clearly observed in the results from the spatial distribution functions.^[^ ^32^ ^]^ Controlling the strength of the intermolecular interactions via component selection and mixing ratio adjustment, maybe a way to suit the demands from different applications.^[^ ^33^ ^]^

DESs with interesting nanostructure, adjustable properties, flammability, high ionic conductivity, and transference number could be excellent ion transport mediums in electrochemical energy storage and conversion systems.^[^ ^12^, ^34^ ^]^ For example, in the mixed lithium bis(trifluoromethanesulphonyl)imide (LiTFSI) and N‐methylacetamide (NMAc), the (N‐H…O) hydrogen bond in NMAc was weakened or even broken by the interaction between LiTFSI and NMAc, resulting in the formation of LiTFSI‐NMAc eutectic electrolyte.^[^ ^35^ ^]^ LiTFSI‐MAc eutectic electrolyte showed a nice room temperature conductivity up to 1.35 mS cm^−1^, and a wide operating temperature range from −72 to 240 °C. Besides, adjusting the nanostructure to construct the stability and facility of the electrolyte–electrode interface was a strategy to realize a high‐performance rechargeable battery.^[^ ^12^ ^]^ These results indicated there was a huge space to explore eutectic electrolytes for energy storage and conversion.

**Figure 2:**
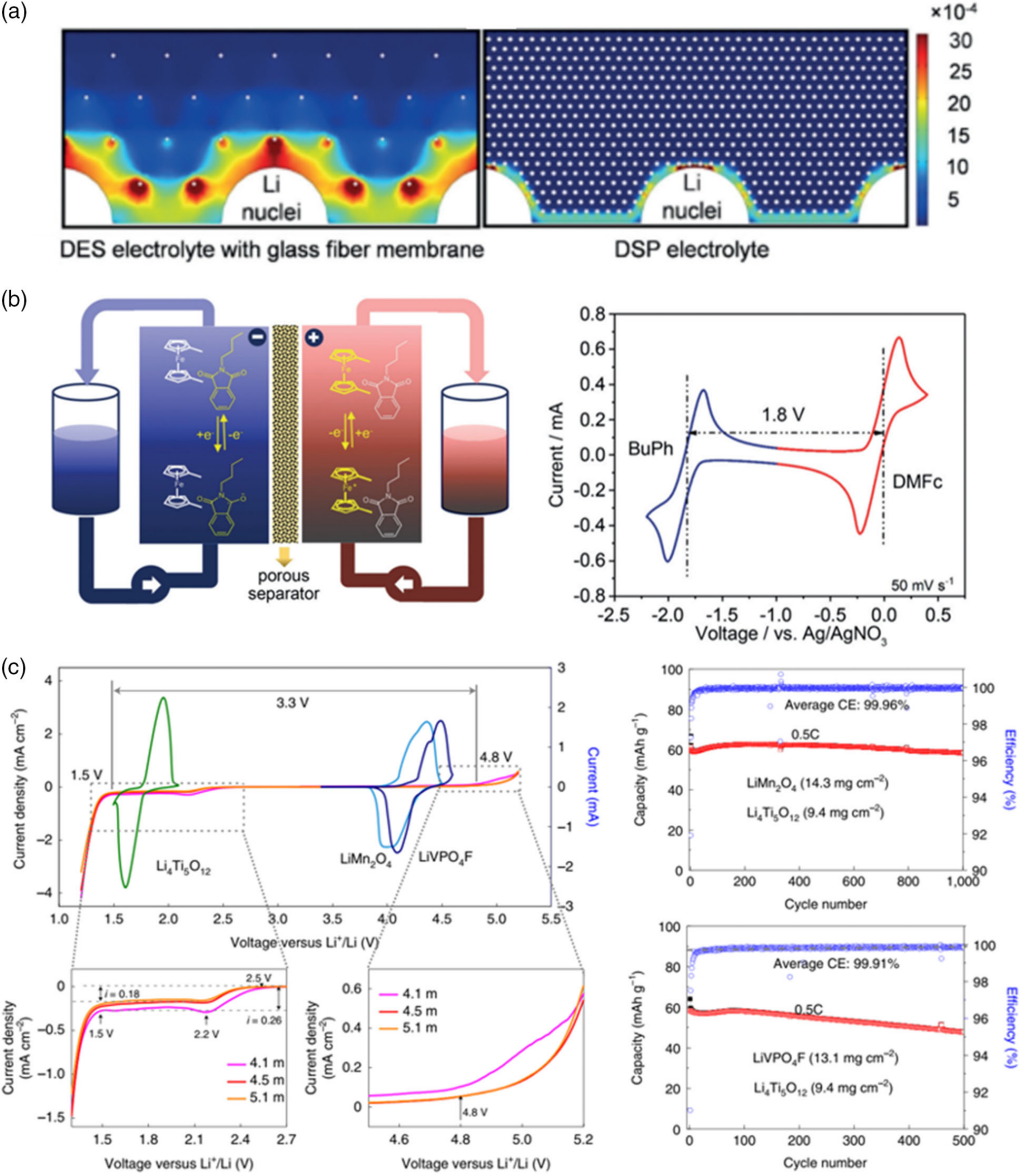
The application of DESs in energy storage and conversion. a) The Li‐ion flux distribution on the interface between Li metal anode and DES or DSP electrolyte via COMSOL. Reproduced with permission.^[^ ^36^ ^]^ Copyright 2020, Wiley‐VCH. b) The schematic redox flow battery assembled with redox‐active DES electrolyte and the CV curve of redox‐active DES electrolyte. Reproduced with permission.^[^ ^37^ ^]^ Copyright 2019, Wiley‐VCH. c) The electrochemical stability window of LiTFSI–KOH–CO(NH_2_)_2_–H_2_O ternary eutectic electrolyte and the cycling stability of full cell in ternary eutectic electrolyte. Reproduced with permission.^[^ ^38^ ^]^ Copyright 2022, Springer Nature.

Rational modifications of composition are important to the design of eutectic electrolyte, which could improve the performance and optimize the interface between electrolyte and electrode.^[^ ^34^ ^]^ For instance, Jaumaux et al. synthesized self‐healing polymer by pentaerythritol tetra‐acrylate (PETEA) and 2‐(3‐(6‐methyl‐4‐oxo‐1,4‐dihydropyrimidin‐2‐yl)ureido)ethyl methacrylate (UPyMA) via thermally polymerizing.^[^ ^36^ ^]^ Then, LiTFSI‐NMAc and fluoroethylene carbonate (FEC) were added to the self‐healing polymer to form a DSP electrolyte. The introduction of FEC could promote the formation of SEI film and protect the anode against Li dendrite growth. Meanwhile, the self‐healing UPyMA‐PETEA copolymer was able to not only prevent the dissolution of Mn^2+^ from LiMn_2_O_4_ cathode into the electrolyte, but also maintain a nice contact between electrode and electrolyte without interfacial cracks during cycling. The simulation results of COMSOL demonstrated that compared to DES + FEC with an inhomogeneous Li^+^ flux, a more regular and orderly distribution of Li^+^ flux could be observed in the DSP electrolyte, which was beneficial to the lithium deposition process (**Figure** **2**a). The strategy to design eutectic electrolytes provided a nice route to develop highly safe and durable rechargeable batteries.

In addition to using as ion transportation or ion supply mediums, DESs composed of the component with redox activity were able to build energy storage devices.^[^ ^39^, ^40^ ^]^ For example, BEE eutectic electrolyte prepared by mixing N‐butylphthalimide (BuPh) and 1,1‐dimethylferrocene (DMFc), was used as the sole electrolyte for the nonaqueous redox flow battery. BuPh/BuPh^−^ redox couple and DMFc/DMFc^+^ redox couple would occur in the redox reaction at the anode and cathode, respectively (Figure 2b).^[^ ^37^ ^]^ Due to the high active specie concentration and high operating voltage, the theoretical energy density of redox flow battery assembled by BuPh‐DMFc electrolyte could achieve 84.4 Wh L^−1^. Meanwhile, the peak power density could reach 192 mW cm^−2^, when operated at 190 mA cm^−2^. These results showed that eutectic electrolyte with redox activity components has demonstrated several unique advantages in building higher energy density and lower prices for next‐generation redox flow batteries.

Moreover, the extremely strong hygroscopic of DESs should be noted. The presence of water could decrease the density and viscosity, and increase the ion conductivity. Besides, the intermolecular peculiar interactions and the microstructure could be retained, which induce unexpected and interesting results.^[^ ^41^, ^42^, ^43^ ^]^ To compete with commercial organic electrolytes, the salt concentration of water in salt electrolyte should be reduced.^[^ ^44^ ^]^ Wang et al. reported a LiTFSI–KOH–CO(NH_2_)_2_–H_2_O ternary eutectic electrolyte, and successfully reduced the salt concentration to 4.5 m.^[^ ^38^ ^]^ In addition, TFSI^−^ and CO(NH)_2_ could generate LiF and polymer layer via reducing at anode. This would lead to the formation of a robust inorganic/organic mixed solid electrolyte interphase, which could expand the electrochemical stability window (>3.3 V) and be beneficial for the stability of battery (Figure 2c). The Li_1.5_Mn_2_O_4_ || Li_4_Ti_5_O_12_ pouch cells using ternary eutectic electrolyte with a low positive/negative capacity ratio of 1.14, exhibited high average Coulombic efficiency (99.96%) and good capacity retention (92% after 470 cycles at an area capacity of 2.5 mAh cm^−2^ at 1C).

## 3 PILs

**Figure 3:**
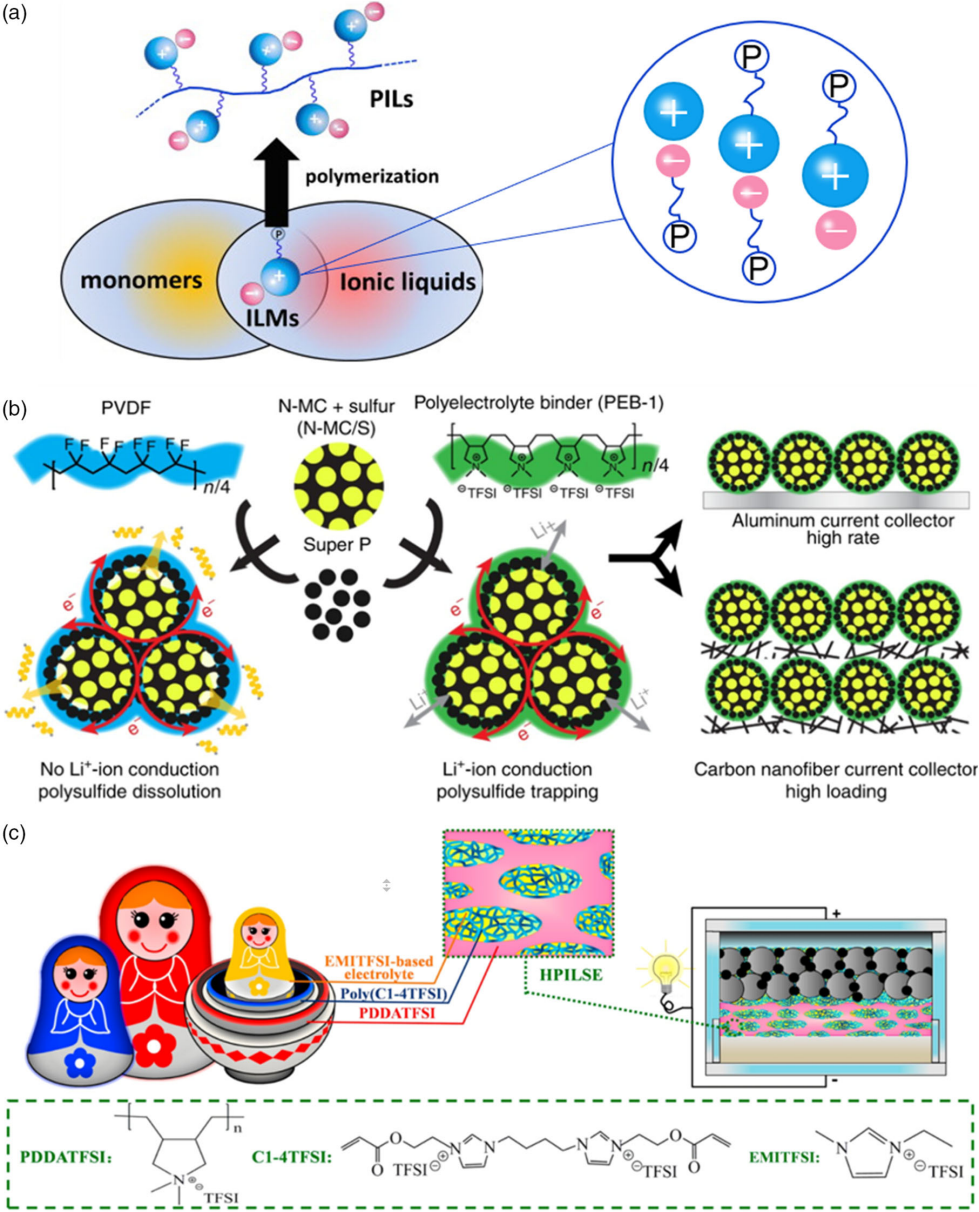
Schematic diagram of poly(ionic liquid)s (PILs) and their related applications in rechargeable batteries. a) Illustration of IL monomers and PILs. “P”: polymerizable group. Reproduced with permission.^[^ ^45^ ^]^ Copyright 2013, Elsevier B.V. b) Scheme of using PVDF or PEB‐1 binder to prepare the sulfur electrode. Reproduced under the terms of the CC‐BY 4.0 license.^[^ ^54^ ^]^ Copyright 2017, The Authors, published by Springer Nature. c) Description of nesting doll‐like hierarchical poly(ionic liquid)‐based solid electrolyte (HPILSE). Reproduced with permission.^[^ ^55^ ^]^ Copyright 2017, Elsevier B.V.

PILs have been synthesized by the polymerization of IL monomers, which are formed by introducing polymerizable units into the anions, cations, or both of them (**Figure** **3**a).^[^ ^45^, ^46^ ^]^ Owing to the polymerization process, the PILs have unique structure and properties that traditional ILs cannot achieve. That means PILs have the potential to fabricate lots of complex and multifunctional materials that are difficult for traditional ILs to prepare. PILs have been showing great potential applications for energy storage and conversion devices, including rechargeable batteries,^[^ ^47^ ^]^ redox flow batteries,^[^ ^48^ ^]^ supercapacitors,^[^ ^49^, ^50^ ^]^ high‐temperature proton exchange membrane fuel cells (HT‐PEMFCs),^[^ ^51^, ^52^ ^]^ electrocatalysis,^[^ ^53^ ^]^ etc.

For example, the addition of the PILs in electrode or electrolyte can help construct the lithium‐ion transport channel and improve the performance of lithium batteries.^[^ ^56^, ^57^ ^]^ A lot of studies have been carried out to utilize the advantages. Recent research in this field has shown the potential of PILs as a binder to replace the polyvinylidene difluoride (PVDF) in LIBs.^[^ ^58^, ^59^ ^]^ In these works, different PILs with the same anion—TFSI^−^, were synthesized. And the LIBs using PILs as binder had excellent performance with an enhanced specific capacity and outstanding cycle stability.

Li–S battery is an important type of high‐energy‐density rechargeable batteries^[^ ^60^, ^61^ ^]^ and the PILs have shown some interesting applications in this field. ^[^ ^62^, ^63^, ^64^ ^]^ Li et al. designed a PIL named poly[(N,N‐diallyl‐N,N‐dimethylammonium) bis(trifluoromethanesulfonyl)imide] (PEB‐1) as binder to fabricate the sulfur electrode in Li–S battery (Figure 3b).^[^ ^54^ ^]^ In this work, by adding the PEB‐1, the hopping of Li^+^ along the weakly bound and mobile anions connected to the polymer backbones was successfully achieved, which promoted the transportation of lithium ions. Besides, they also found that the formed polysulfides had the preference to replace the TFSI^−^ and connect to the polycation backbone at their anionic terminus. In this case, the transportation of polysulfides was regulated and their diffusion from the carbon hosts was limited. This phenomenon was further confirmed by both X‐ray photoelectron spectroscopy (XPS) and density functional theory (DFT). As a result, the batteries exhibited excellent performance. Even in the case of high sulfur loads up to 8.1 mg cm^−2^, the batteries maintained high utilization of sulfur and lower capacity fade. Based on these findings, Vizintin and coworkers further explored the effects of different cations on performance and found that the styrene backbones and crosslinking structure in the PILs would lead to better performance.^[^ ^64^ ^]^

Besides, solid state and nice ionic conductivity also allow PILs as promising materials for solid‐state electrolyte. Some related research have been carried out to confirm their practical applications in energy devices, and the structure–properties relationships have been also elaborated by far.^[^ ^65^, ^66^, ^67^, ^68^ ^]^ For example, Zhou et al. prepared a nesting doll‐like hierarchical poly(ionic liquid)‐based solid electrolyte (HPILSE) to overcome the low ionic conductivity and poor mechanical properties (Figure 3c).^[^ ^55^ ^]^ The HPILSE exhibited satisfying ionic conductivity (above 10^−3^ S cm^−1^ at 25 °C) and tensile strength (2.4 MPa). When testing in LiFePO_4_/Li‐HPILSE/Li cell, the cell showed excellent cycling stability with a capacity retention of 97.7% at 0.1C after 100 cycles. And the Coulombic efficiency was close to 100% in this process except for the first cycle. When used in the Na‐ion cell, the same result could be gotten with a capacity retention of 85.5% and a Coulombic efficiency close to 100%. Forsyth's group found the introduction of polycations could limit the diffusion of anions, leading to an enhanced Li^+^ transport number in the solid‐state electrolyte based on the PILs.^[^ ^69^, ^70^, ^71^, ^72^ ^]^ Moreover, they also found the addition of Li salt in PILs would lead to the breaking of the strong anion–polycation interaction, and the FSI^−^ anion would co‐coordinate with both the Li^+^ and the polycation. Owing to these features, the transportation of Li^+^ was facilitated. These findings would be important for the construction of high‐safety rechargeable Li‐ion batteries.^[^ ^69^ ^]^

In addition to the applications in rechargeable batteries, the electrolyte derived from PILs can be also used for supercapacitors.^[^ ^73^, ^74^, ^75^, ^76^ ^]^ Marcilla et al. reported the all‐solid‐state supercapacitors (SCs) based on PIL/IL binary blend for the first.^[^ ^49^ ^]^ The SCs could operate at voltages as high as 3.5 V and the specific capacitance and real energy could reach 100 F g^−1^ and 32 Wh kg^−1^ separately at 1 mA cm^−2^. This was an excellent attempt to use PILs‐based solid‐state electrolytes in supercapacitors. And after that, other related studies have been also reported.^[^ ^77^, ^78^ ^]^

**Figure 4:**
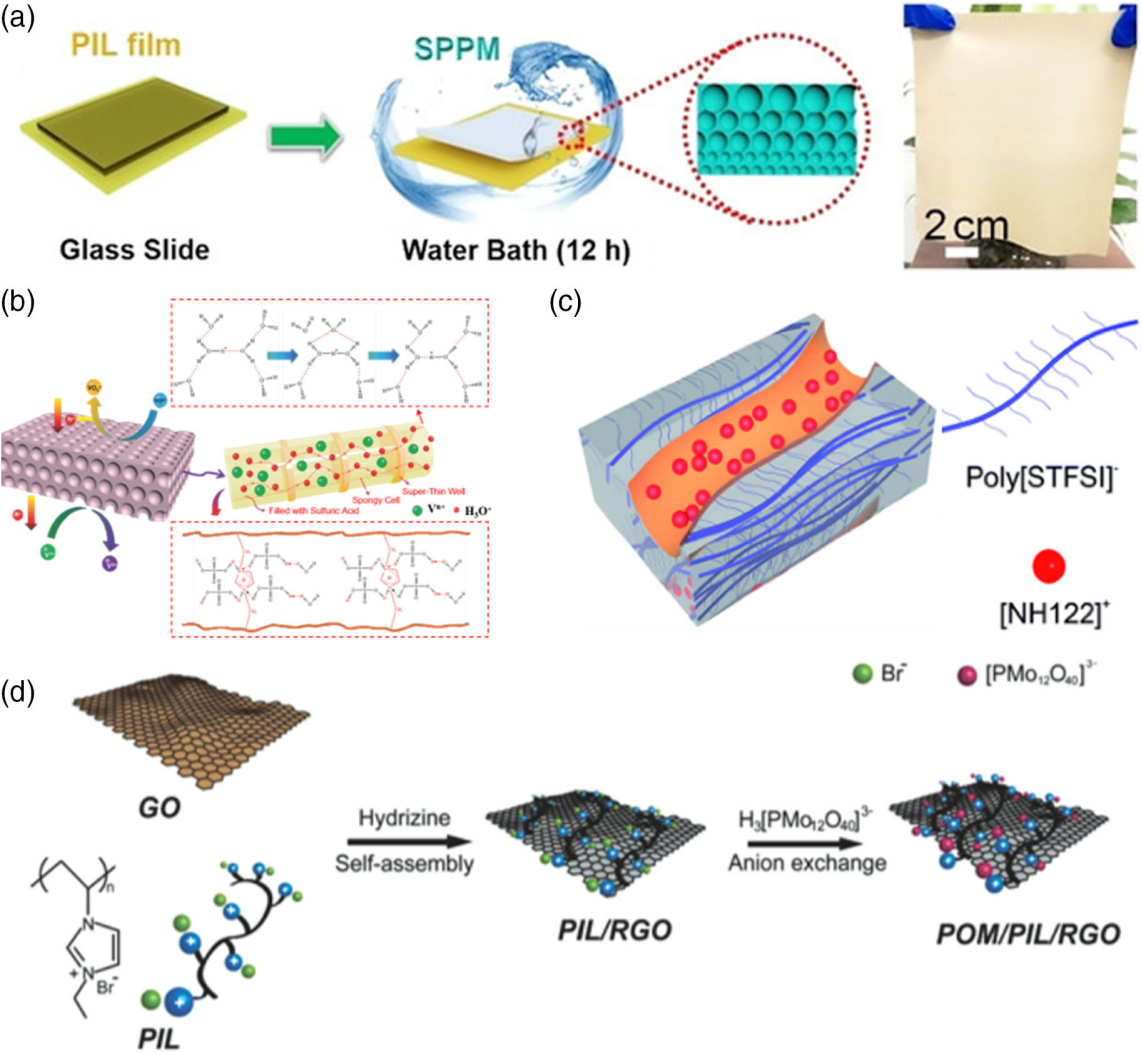
Other applications of PILs and their related self‐assembly behavior. a) The fabrication process of supramolecular porous polyelectrolyte membranes (SPPMs). Reproduced with permission.^[^ ^79^ ^]^ Copyright 2020, Wiley‐VCH. b) Schematic diagram of the charged sponge‐like porous membranes. Reproduced with permission.^[^ ^48^ ^]^ Copyright 2016, Wiley‐VCH. c) Self‐assembled nanostructure of the protic PILs. Reproduced with permission.^[^ ^82^ ^]^ Copyright 2016, Royal Social of Chemistry. d) Principle of the formation of POM/PIL/RGO. Reproduced with permission.^[^ ^83^ ^]^ Copyright 2014, Wiley‐VCH.

There are also applications of PILs in the preparation of membranes or electrodes. For example, in recent studies, Shao and coworkers successfully constructed four supramolecular porous polyelectrolyte membranes (SPPMs) by using four different PILs (**Figure** **4**a).^[^ ^79^ ^]^ In this process, they used water as an H‐bonding cross‐linker to induce the microphase separation of the hydrophilic cation ring from the hydrophobic network. As a result, the pores were formed. In this work, they proposed a new and simple method to fabricate adjustable porous membranes from PILs and proved their potential possibilities to fit different applications. The practical applications of PILs‐based membranes in energy storage and conversion have been also studied. For instance, Liu et al. used a cross‐linkable polymeric ionic liquid (cPIL) as a crosslinker and prepared a new type of cross‐linked composite membrane successfully.^[^ ^80^ ^]^ Compared with the linear structure, the cross‐linked network could limit phosphoric acid leaking. This kind of membrane had better stability, mechanical properties, and a higher PA doping level. In the latter work, they also further explained that the high proton conductivity in the PBI/PIL composite membrane stemmed from the synergy of the Grotthuss mechanism and the vehicle mechanism.^[^ ^81^ ^]^ Moreover, Zhao et al. prepared a charged sponge‐like porous membrane and used it in vanadium redox flow batteries (Figure 4b).^[^ ^48^ ^]^ Due to the Donnan exclusion from the positively charged cations, the vanadium could be effectively retained, which resulted in high selectivity. The crosslinked network also enhanced the stability. These features led to a Columbic efficiency of 99% and an energy efficiency of 86% at the current density of 80 mA cm^−2^ for a single cell. And the vanadium flow batteries (VFBs) using this membrane could maintain stability for more than 6000 cycles.

In addition, some researchers also used PILs as structural elements to prepare ordered materials via a bottom‐up self‐assembly approach and used them in energy storage and conversion. For example, Cordella et al. synthesized block copolymers(BCPs) based on amphiphilic double poly(N‐vinyl imidazolium) by self‐assembly.^[^ ^84^ ^]^ These BCPs had the ionic conductivity of 1–3 × 10^−7^ S cm^−1^ at 30 °C under nonaqueous condition and showed a wide electrochemical window (≈4.8 V vs. Li^+^/Li). Besides, the free‐standing membranes with proper mechanical performance could be also formed by using these BCPs. And owing to these features, the membranes were promising for the applications of single‐ion solid polyelectrolytes. Shah and coworkers also fabricated a free‐standing membrane consisting of protic PILs, poly([NH122][STFSI]), through the solvent casting method (Figure 4c).^[^ ^82^ ^]^ These PILs were composed of polymeric and highly dissociable anions. And at the microscopic scale, the dissociated cations, [NH122]^+^, could act as proton carriers and construct nano‐scale interconnecting channels by self‐assembling, leading to a fast proton transport of about 3.1 × 10^−4^ S cm^−1^ at 120 °C. Moreover, Yang et al. used polyoxometalates (POMs), PILs, and graphene oxide (RGO) as structural units to prepare POM/PIL/RGO nanohybrids via self‐assembly and anion exchange method (Figure 4d).^[^ ^83^ ^]^ In this process, the PILs acted as an interfacial linker and could make the distribution of POMs more uniform. Besides, they also found the addition of PILs could build proton transfer channels and promote the charge transport at the interface, leading to an enhanced redox reaction of POMs. As a result, the supercapacitors with POM/PIL/RGO electrodes had a higher specific capacitance, energy, and power density and a longer cycling life compared to those with POM/RGO electrodes.

## 4 ILCs

LCs are a class of materials with ordered structure via self‐assembly, which are thermodynamically located between the fluids and the 3D solid crystal. ILCs are emerging materials, that truly combine the functional properties of ILs (ionic character and physically/chemically dynamic natures) and liquid crystals (anisotropy, fluidity, and a self‐assembling nature).^[^ ^17^, ^18^ ^]^

**Figure 5:**
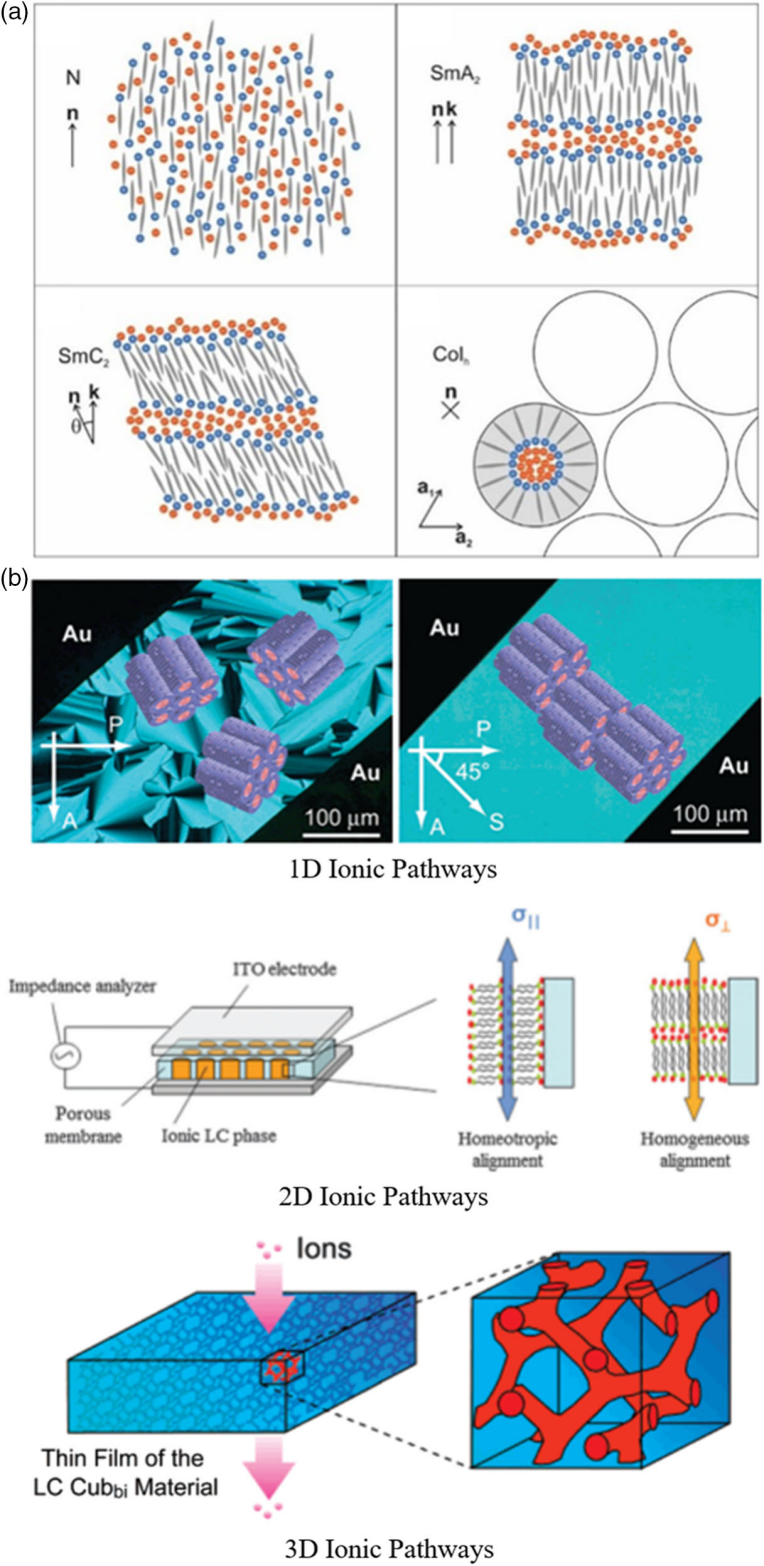
Self‐assembly for structures and nanochannels. a) The ion‐rich regions of ILCs promoted by nanosegregation, and lead to different phase behavior to nonionic LCs. Reproduced with permission.^[^ ^85^ ^]^ Copyright 2021, Wiley‐VCH. b) Building different nanostructures of ILCs for ions conduction at 1D,^[^ ^86^ ^]^ 2D,^[^ ^87^ ^]^ and 3D^[^ ^88^ ^]^ via adjusting the size, shape, and charge distribution of component. Reproduced with permission.^[^ ^86^ ^]^ Copyright 2004, American Chemical Society. Reproduced with permission.^[^ ^87^ ^]^ Copyright 2015, Royal Society of Chemistry. Reproduced with permission.^[^ ^88^ ^]^ Copyright 2007, American Chemical Society.

Nanosegregation caused by contrasts in the molecules is the primary driver of the formation of ILCs in the self‐assembly process. The compatible structurally and/or chemically parts and the chemical bonding between the incompatible parts result in the formation of different molecular regions at the microscale. The incompatibility between hydrophilic and hydrophobic parts is a typical example. Compared with nonionic LCs, the presence of charges leads to strong noncovalent interactions between ions via Coulombic interactions, increasing the degree of amphiphilicity of LCs. The nanosegregation of nonionic parts and ionic parts is reinforced and the formation of ion‐rich regions is promoted.^[^ ^85^ ^]^ Therefore, the distribution of ions is difficult to disperse uniformly in the bulk, it tends to form lamellar or columnar structure, which aggravated the difference between the ionic region and nonionic region. The combination of differential scanning calorimeter, polarizing microscope, and small angle X‐Ray scattering could analyze the nanostructures of ILCs and confirm this phenomenon. The results showed that the nematic (N) phase with a relatively uniform distribution of ions and SmC phase with a consistent tilt angle in different layers, were rarely observed in ILCs (**Figure** **5**a). ILCs tend to form SmA phase and columnar mesophases, which is different from many nonionic LCs.

Owing to these characteristics, ILCs with smectic structure^[^ ^87^ ^]^ and cubic structure^[^ ^88^, ^89^ ^]^ were synthesized to realize 1D, 2D, and 3D ion‐rich nanochannels (Figure 5b) and achieve more efficient ions transportation. For example, Yoshio and coworkers reported a new ILC with a self‐organized columnar, physically continuous, and highly ordered ion‐rich region.^[^ ^86^, ^90^ ^]^ The ion conduction in parallel to the columnar axis was higher than those perpendicular to the axis. The results proved that ILCs were able to achieve 1D anisotropic ionic conductivities. Moreover, some studies have proved the phase structure changes of ILCs are crucial to ion conductions. Soberats et al. reported a benzenammonium columnar ILC with thermoreversible phase transition.^[^ ^91^ ^]^ The ILC could transform the structure from the rectangular columnar (Col_r_) into hexagonal columnar (Col_h_) phases via heating, and the corresponding ion conductives were increased four orders of magnitude. Further work has been done to control the macroscopic order of the nanosegregated ILC structures via the design in the size and shape of molecules.^[^ ^87^, ^88^, ^92^, ^93^ ^]^ All of these studies have demonstrated that ILCs with efficient and directional ions transportation has been a class of promising material for biological processes and advanced device technologies.

**Figure 6:**
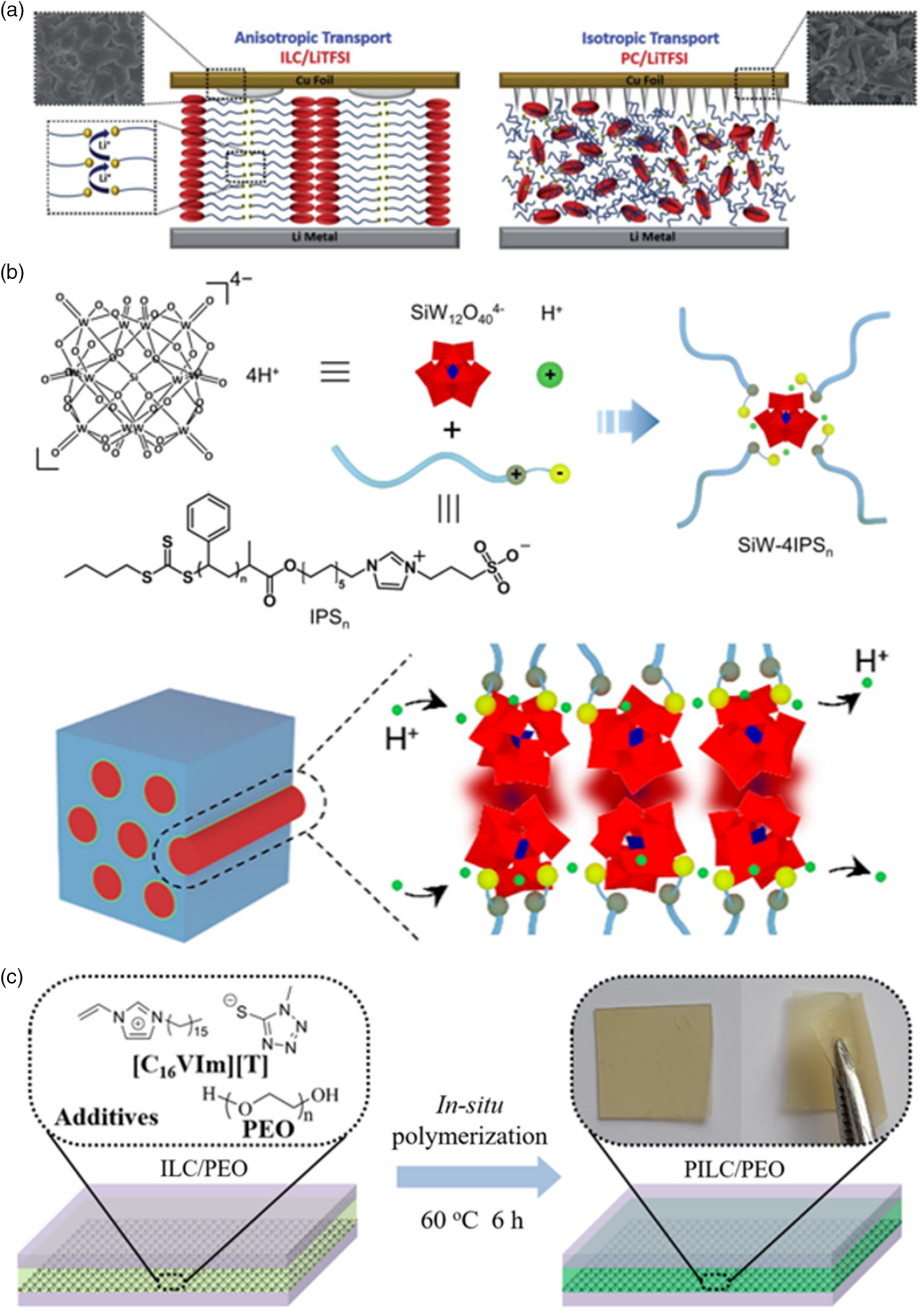
The application of ILCs in energy storage and conversion. a) Li deposition schematic of the anisotropic transport and the isotropic transport. Reproduced with permission.^[^ ^97^ ^]^ Copyright 2021, The Royal Society of Chemistry. b) The Fabrication of Polyoxometalate‐Based Liquid‐Crystalline Electrolyte SiW‐4IPSn and the Formation of Its Columnar Nanophase. Reproduced with permission.^[^ ^98^ ^]^ Copyright 2021, American Chemical Society. c) Compositions and procedure for all‐solid‐state electrolyte PILC/PEO preparation within a DSSC. Reproduced with permission.^[^ ^99^ ^]^ Copyright 2021, American Chemical Society.

For energy storage and conversion, compared to liquid electrolytes with isotropic ionic conduction, the anisotropic ILCs with good electrochemical windows and appreciable conductivity, may open a new way to build high‐performance energy storage devices.^[^ ^94^, ^95^, ^96^ ^]^ Arava et al. reported a novel ILC electrolyte composed of imidazolium‐based thermotropic ILC material and LiTFSI.^[^ ^97^ ^]^ The DSC measurement showed that the ILC‐LITFSI mixture electrolyte with 9:1 molar ratio maintained SmA phase in a wide temperature range of 14–140 °C. The ILC‐LITFSI mixture electrolyte showed a nice ion conductivity of 1.85 × 10^−4^ S cm^−2^ at a temperature of 60 °C. Moreover, compared to PC‐LiTFSI isotropic electrolyte with sharp needle‐like deposits, Li deposited in ILC/LiTFSI exhibited spherical‐like morphology that corresponded to the formation of Li dendrites (**Figure** **6**a). The anisotropic ion transportation of ILCs could suppress dendrite growth without sacrificing battery performance. These results indicated that ILCs were a class of promising electrolyte materials to be used as solid‐state electrolytes with fast ion‐conductive tunnels for electrochemical energy devices.

In addition to Li‐ion conduction, ILCs with well‐defined molecular structures and ordered nanochannels are also regarded as one of the most promising materials to achieve efficient proton conduction.^[^ ^87^, ^100^ ^]^ For instance, Chai et al. reported a thermotropic ILC electrolyte by the self‐assembly of a polyoxometalate cluster H_4_SiW_12_O_40_ (SiW) and an amphiphilic zwitterion‐terminated polymer ligand (Figure 6b).^[^ ^98^ ^]^ The zwitterionic consisted of an imidazole ring and a sulfonate group, and created a system with well‐defined microphase separation. Moreover, there were strong interactions between SiW anions and the imidazole ring, while the protons of SiW were captured by the sulfonate group. These phenomena promoted the construction of a well‐ordered hexagonal columnar phase and SiW‐based columnar nanochannels, which could operate at a wide temperature range from 90 to 160 °C. The proton conductivity of the ILC electrolyte attained 3.4 × 10^−2^ mS cm^−1^ under the temperature of 160 °C. The results demonstrated that ILC‐based materials with highly stable nanochannels and nice anhydrous proton conduction would be an excellent complementary to Nafion at high‐temperature conditions. With further improved proton‐conducting efficiency, mechanical toughness, and electrochemical stability, there is the promise of using ILCs to build advanced proton transport systems.^[^ ^101^ ^]^

ILCs are also able to serve as ideal materials to construct dye‐sensitized solar cells (DSSCs).^[^ ^24^, ^102^ ^]^ ILCs as a possible alternative material could locally concentrate the couple, and enhance the exchange reaction and hole transportation. The liquid crystalline phase could realize a good balance between dye regeneration and hole transport. For example, Zhou et al. mixed a smectic ILC containing the vinylimidazolium thiolate/disulfide redox group and polyethylene oxide (PEO) to in situ polymerized PILC/PEO solid‐state electrolyte (Figure 6c).^[^ ^99^ ^]^ The microphase segregation nanostructure with efficient charge transport was preserved. Due to the fluidity of ILC molecules, PILC/PEO maintained a nice conductivity of 3 × 10^−5^ S cm^−1^ (25 °C) to 7 × 10^−4^ S cm^−1^ (70 °C) and made good interfacial contact in DSSC. In simulated AM1.5 G solar light, the PCE of DSSC‐PILC/PEO had reached 4.0% at 60 °C. Although the efficiency and durability of current ILC‐based DSSCs were relatively poor, this novel electrolyte had different advantages in constructing solid‐state DSSC with interfacial contacts and efficient transportation.

## 5 RAILs

The electro‐active molten salts have been already reported early in the 1990s.^[^ ^103^, ^104^ ^]^ These efforts lay the foundation for latter research on RAILs. RAILs mainly consist of two types.^[^ ^105^ ^]^ One is based on counter ions with intrinsic redox activity, including iodide ions,^[^ ^106^, ^107^ ^]^ viologen ions,^[^ ^108^, ^109^, ^110^ ^]^ and others.^[^ ^111^, ^112^ ^]^ The other consists of anions or cations modified by redox groups, such as Fc,^[^ ^113^, ^114^, ^115^ ^]^ TEMPO,^[^ ^116^, ^117^ ^]^ metal complexes,^[^ ^118^ ^]^ etc. This means RAILs are not only ionically conductive and fluid, but also have concentrated and adjustable redox centers. Based on these unique features, electrochemical reactions can be carried out without additional electrolyte or solvent. At present, RAILs have been showing excellent potential applications for energy storage and conversion.

**Figure 7:**
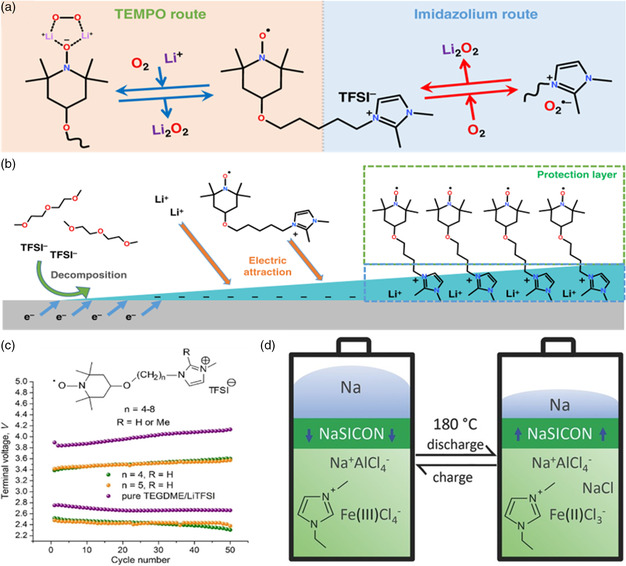
The application of PILs in rechargeable batteries. a,b) Schematic illustration of roles of the IL‐TEMPOs in Li–O_2_ batteries. Reproduced under the terms of the CC‐BY 4.0 license.^[^ ^124^ ^]^ Copyright 2019, The Authors, published by Springer Nature. c) The cycle performance of using different IL‐TEMPOs in Li–O_2_ batteries. Reproduced with permission.^[^ ^116^ ^]^ Copyright 2020, American Chemical Society. d) Principle of the Na‐Fe battery based on EMIFeCl_4_‐NaAlCl_4_ catholyte and liquid sodium anolyte. Reproduced with permission.^[^ ^125^ ^]^ Copyright 2015, Wiley‐VCH.

Rochefort and coworkers have carried out some research about RAILs.^[^ ^119^, ^120^, ^121^, ^122^, ^123^ ^]^ For example, ferrocene‐functionalized imidazolium RAILs were first used as redox shuttles and successfully prevented the overcharging of the batteries.^[^ ^123^ ^]^ In addition, due to the miscibility of RAILs with ethylene carbonate–diethyl carbonate solvent, a high concentration of redox shuttles could also be achieved. Their works provided a possible strategy to achieve highly concentrated redox shuttles in lithium‐ion batteries. Besides, Zhang and coworkers introduced a RAIL additive based on 2,2,6,6‐tetramethylpiperidine‐1‐oxyl (TEMPO), IL‐TEMPO, and used it in Li‐O_2_ batteries.^[^ ^124^ ^]^ In this system, due to the redox‐active TEMPO groups and the positively charged imidazole groups, the IL‐TEMPO could interact with O_2_, O_2_^−^, which promoted the dissolution of oxygen and suppressed the by‐reaction at the same time, leading to an enhanced discharge capacity and a lower charge voltage. Besides, the imidazole moiety of IL‐TEMPO could also be attached to a lithium metal anode. This process led to the formation of a more stable SEI layer and increased the ion transfer at the interface, which protected the lithium metal anode and allowed the lithium to be stripped and plated smoothly. Owing to these features, the RAIL could serve as multifunction including redox mediators, oxygen shuttles (**Figure** **7**a), and lithium anode protector **(**Figure 7b) at the same time, and improved the cycle life and performance of Li‐O_2_ batteries from all aspects. In their latter research, they further elucidated the effects of substituents (R = H or Me) in the imidazole ring and the number of alkyl chain carbon atoms (*n* = 4–8) (Figure 7c).^[^ ^116^ ^]^ In this study, they found when R = H, the compounds exhibited better ionic conductivity, which led to better performance. They also found that Li–O_2_ had better performance when *n* = 4, 5. And after 50 cycles, the charge overpotential was reduced by ≈0.6 V.

Angell et al. also successfully assembled a Na‐Fe cell based on a redox IL—EMIFeCl_4_.^[^ ^125^ ^]^ In this cell, the EMIFeCl_4_ served as the joint ion‐conducting medium and liquid cathode material, while the anode was composed of liquid sodium and was separated from the catholyte material by a sodium ion‐conducting ceramic separator (NaSICON) (Figure 7d). When testing in the full cell at 180 °C, the cell exhibited good performance with a high voltage above 3.2 V and high energy efficiency above 96%. Besides, even if the cathode and anode came into contact with each other, there was no gas produced, which avoid the occurrence of an explosion. This work provided a new idea for the application of RAILs.

Moreover, RAILs can also be used as functional electrolytes in electrochemical supercapacitors. In pioneering works, researchers found an increasing capacitance resulting from both the traditional EDL capacitance and the pseudo‐capacitance from the redox reaction of the RAILs.^[^ ^106^, ^122^, ^126^ ^]^ For instance, Fontaine et al. groundbreakingly used a new type of IL, biredox IL, in supercapacitors, whose anions and cations were both functionalized with redox‐active moieties.^[^ ^127^, ^128^, ^129^ ^]^ The introduction of biredox centers made the redox species concentration of the ILs close to that of the solid state, which endowed them a solid‐state capacity and a liquid‐state reaction kinetics as while. In one of their representative studies, they used anthraquinone (AQ) and 2,2,6,6‐tetramethylpiperidinyl‐1‐oxyl (TEMPO) to functionalize the anions and cations, respectively.^[^ ^130^ ^]^ The RAILs were used in electrochemical double layer capacitors (EDLC). When charging the capacitor, the redox‐active AQ‐PFS^−^ and BMIm^+^‐TEMPO^−^ were adsorbed on the surface of the electrode and then undergone fast Faradaic reactions, leading to a doubled capacitance. Besides, the RAILs could also be trapped in the pores of the electrode due to their large size, which prevented them from diffusing to the other electrode. As a result, the self‐discharge and leakage current decreased.

**Figure 8:**
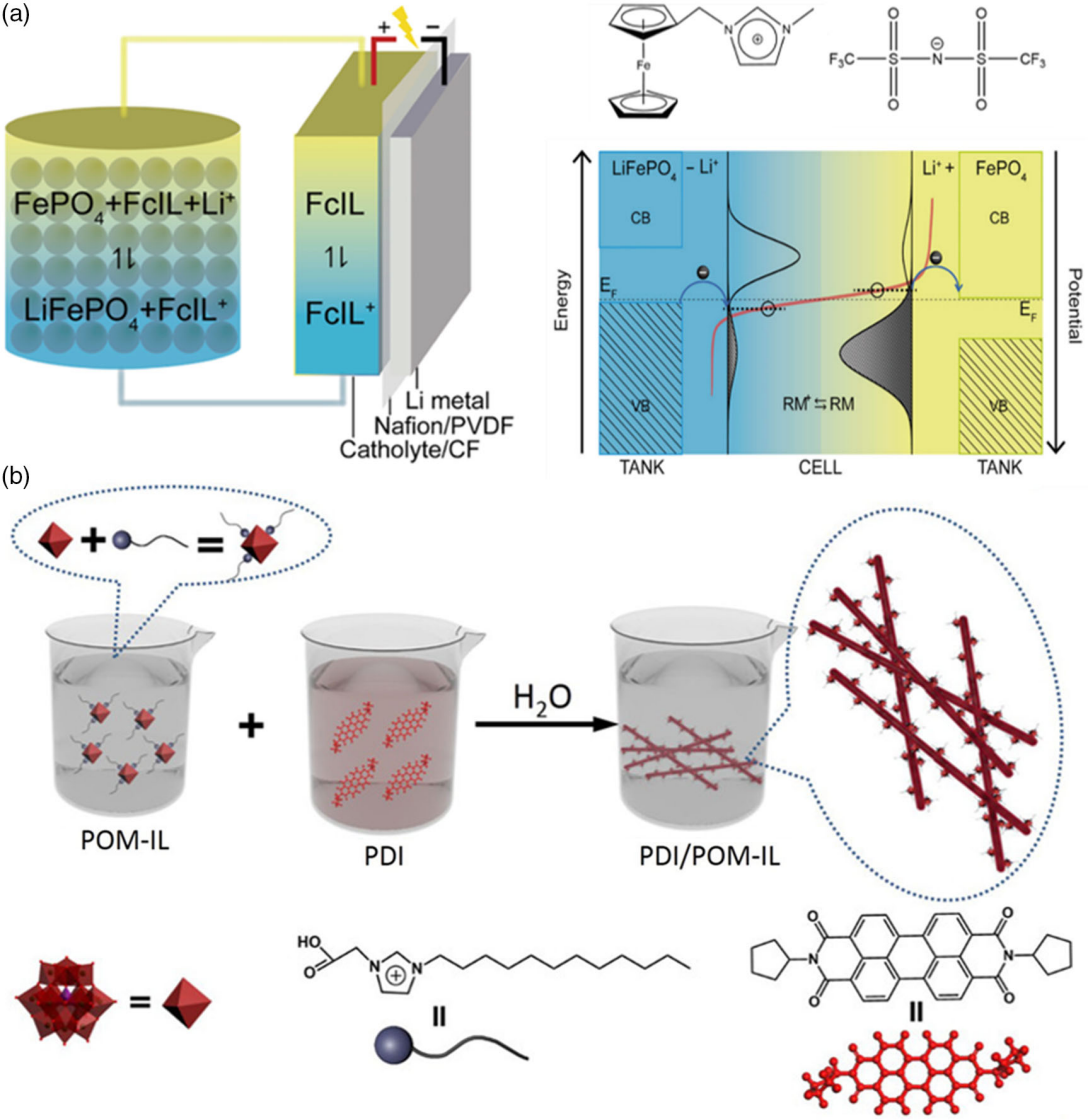
The application of RAILs in other fields of energy storage and conversion. a) Illustration of the FcIL‐based redox flow lithium batteries (RFLBs) and operating principle of single‐molecule redox‐targeting reaction. Reproduced with permission.^[^ ^133^ ^]^ Copyright 2017, Elsevier B.V. b) Illustration of the preparation of PDI/POM‐IL nanofibers via self‐assembly. Reproduced with permission.^[^ ^134^ ^]^ Copyright 2021, American Chemical Society.

In addition, the RAILs have also shown great potentiality in redox flow batteries. Anderson et al. proposed a method to increase the energy density of RFBs by increasing the concentration of the redox centers.^[^ ^131^ ^]^ In their work, RAILs, named MetILs^3^, with multiple redox centers in cation core, ligands, and anions were prepared. This kind of RAILs possessed a high concentration of redox‐active electrons in practice up to 4.2 M, 2.3 times higher than the MetIL they prepared originally.^[^ ^132^ ^]^ This method led to higher capacity. Zhou et al. also proposed another strategy.^[^ ^133^ ^]^ They used ferrocene‐grafter ionic liquids (FcILs) as intermediates to design redox flow lithium batteries (RFLBs) based on single‐molecule redox‐targeting (SMRT) reaction (**Figure** **8**a). In the RFLBs, the redox couples FcILs and LiFePO_4_/FePO_4_ had the same standard potential. When charging or discharging, the Nernst potential of both couples changed, leading to the formation of a driven force. This driven force promoted the migration of electrons, which facilitated the reversible delithiation and lithiation of LiFePO_4_. As a result, the voltage efficiency reached up to 95%. The energy density was also enhanced to 330 Wh L^−1^, about ten times of a vanadium flow battery.

Polyoxometalates (POMs) are nano‐scale metal–oxide clusters composed of pre‐transition metal ions with the highest oxidation state through sharing the oxygen atoms. POMs are usually formed by self‐assembly through a bottom‐up approach, which endows them flexible and adjustable structures. Besides, owing to the presence of surface hydrophilicity and terminal oxygen, most POMs conduct ions well.^[^ ^135^ ^]^ Moreover, the metal ions of the highest state also give POMs superior redox performance. These features broaden the application of POMs and make them good candidates for energy storage and conversion.^[^ ^136^, ^137^, ^138^, ^139^, ^140^, ^141^ ^]^ Recently, a new kind of RAILs has been prepared by replacing the anions of ILs with polyoxometalates, which are described as POM‐ILs.^[^ ^142^, ^143^, ^144^ ^]^ This kind of RAILs combines the properties of both POMs and ILs, which gives them the characteristics of tunable properties and ease of processing. POM‐ILs have shown great potential applications in energy storage and conversion in recent years.^[^ ^145^, ^146^, ^147^ ^]^ For example, Cruz et al. reported four POM‐ILs based on phosphomolybdate anion (PMo_12_O_40_^3−^).^[^ ^148^ ^]^ In this work, the POM‐ILs were adsorbed to the TiO_2_ film and were used as photosensitizers. Zhang et al. synthesized a nanofiber composed of PDI and POM‐IL by hierarchical self‐assembly (Figure 8b).^[^ ^134^ ^]^ In this work, they found the addition of POM‐ILs could lower the bandgap, which promoted visible light absorption of materials. Besides, the POM‐ILs could also accept the photogenerated electrons coming from PDI rapidly and prevent the rapid recommendation of electron–hole pairs. Both of the reasons made the nanofiber an excellent performance in terms of photoelectric response, which meant great potential in the fields of optoelectronics.

## 6 Conclusion and Perspective

The development of ILs is entering an era of functionalization that more and more novel ILs with specific functions have been synthesized. The introduction of novel ILs injects new development vitality and brings people's views away from solvents to other directions. This also broadens their applications in energy storage and conversion to meet the increasing requirements. In this review, the recent advances in novel ILs have been discussed. We have introduced the structures and unique properties of novel ILs briefly. And we have also presented, in detail, the applications of different novel ILs in the fields of energy storage and conversion, such as batteries, fuel cells, supercapacitors as well as redox flow batteries. Nonetheless, it is undeniable that research in this area is still in the exploratory stage. To realize novel ILs for practical applications, there are still many challenges that need to be addressed. 1) Although a large number of DESs have been successfully prepared and the ability to build high‐performance energy storage devices is also proven, the unclear potential connections between the properties and the constituents, as well as the limited cognitions of the interaction and nanostructure, will great impede the continued progress of DESs. Future works should be devoted to developing exact relationships or empirical methods to explain, and even predict these foundations. Moreover, the intrinsic mechanism and the dynamics of DESs used in energy storage devices should be further revealed by in situ/ex situ characterization. 2) Compared to other polymers, PILs have some unique properties like ionic conductivity and electrochemical stability. These endow PILs with great advantages in the preparation of energy materials such as membranes, electrolytes, electrodes, etc. and attract broad interest. Although some results have been achieved, the low ionic conductivity, simple functionality, and ambiguous structure‐properties relationships limit the applications. Further research should be carried out to prepare new ions to improve the performance like conductivity. And relevant studies are still needed to help understand the structure–properties relationships and the effect of different ions on the performance to guide the synthesis of PILs. In addition, developing novel functional PILs, such as hydrophobic PILs and redox‐active PILs, to fabricate new materials is also an important direction. 3) The ionic character and the unique self‐assembly behavior of ILCs allow them to be promising candidates under some complex conditions. It is clear that the future of ILCs is strongly correlated with more experiments, which are devoted to understanding, and even controlling the basic synthetic, structure formation, structure–properties relationships, and tuning phase transitions. Moreover, numerous theoretical investigations are needed to focus on the physical and chemical properties of ILCs, and reveal the role of the constituting ions to influence the phase behavior. With intensive study, developing ILCs with complex phase behavior and more interesting features is possible, and achieves the true versatility of ILCs. 4) RAILs are formed by the introduction of ions with redox‐active properties or the functionalization of ions of ILs with redox‐active groups. The introduction of redox‐active centers broadens the applications of ILs beyond the inert electrolytes and solvents and makes RAILs promising materials in energy systems. However, the introduction of redox‐active groups may lead to an increase in viscosity and a decrease in the decomposition temperature. This will further lead to the deterioration of mass transfer velocity, ionic conductivity, and stability. Besides, the attachment of redox‐active groups to the ions can influence the redox properties, such as redox potential and reversibility. Moreover, the steps to synthesize RAILs are cumbersome, which limits the further applications of RAILs. Thus, strategies to meet the above issues will be important for the further applications of RAILs.

In conclusion, there is still a long way to go to realize the practical applications of novel ILs in electrochemical energy storage and generation systems. And this process requires the efforts of researchers from different fields. Besides, the development of green chemistry requires that the future ILs must be environmentally friendly and degradable as well as low in price. We believe with the deepening of research, more and more novel ILs will be developed and applied in different energy devices practically.

## Conflict of Interest

The authors declare no conflict of interest.
